# Evolving mutation rate advances the invasion speed of a sexual species

**DOI:** 10.1186/s12862-017-0998-8

**Published:** 2017-06-26

**Authors:** Marleen M. P. Cobben, Oliver Mitesser, Alexander Kubisch

**Affiliations:** 1 Department of Animal Ecology, Netherlands Institute of Ecology (NIOO-KNAW), PO Box 50, 6700 AB Wageningen, The Netherlands; 2 Theoretical Evolutionary Ecology Group, Institute for Animal Ecology and Tropical Biology, University of Würzburg, Emil-Fischerstr. 32, 97074 Würzburg, Germany; 3 Institute for Landscape and Plant Ecology, University of Hohenheim, August-von-Hartmann-Str. 3, 70599 Stuttgart, Germany

**Keywords:** Local adaptation, Spatial sorting, Individual-based model, Evolvability, Dispersal evolution, Metapopulation

## Abstract

**Background:**

Many species are shifting their ranges in response to global climate change. Range expansions are known to have profound effects on the genetic composition of populations. The evolution of dispersal during range expansion increases invasion speed, provided that a species can adapt sufficiently fast to novel local conditions. Genetic diversity at the expanding range border is however depleted due to iterated founder effects. The surprising ability of colonizing species to adapt to novel conditions while being subjected to genetic bottlenecks is termed ‘the genetic paradox of invasive species’. Mutational processes have been argued to provide an explanation for this paradox. Mutation rates can evolve, under conditions that favor an increased rate of adaptation, by hitchhiking on beneficial mutations through induced linkage disequilibrium. Here we argue that spatial sorting, iterated founder events, and population structure benefit the build-up and maintenance of such linkage disequilibrium. We investigate if the evolution of mutation rates could play a role in explaining the ‘genetic paradox of invasive species’ for a sexually reproducing species colonizing a landscape of gradually changing conditions.

**Results:**

We use an individual-based model to show the evolutionary increase of mutation rates in sexual populations during range expansion, in coevolution with the dispersal probability. The observed evolution of mutation rate is adaptive and clearly advances invasion speed both through its effect on the evolution of dispersal probability, and the evolution of local adaptation. This also occurs under a variable temperature gradient, and under the assumption of 90% lethal mutations.

**Conclusions:**

In this study we show novel consequences of the particular genetic properties of populations under spatial disequilibrium, i.e. the coevolution of dispersal probability and mutation rate, even in a sexual species and under realistic spatial gradients, resulting in faster invasions. The evolution of mutation rates can therefore be added to the list of possible explanations for the ‘genetic paradox of invasive species’. We conclude that range expansions and the evolution of mutation rates are in a positive feedback loop, with possibly far-reaching ecological consequences concerning invasiveness and the adaptability of species to novel environmental conditions.

**Electronic supplementary material:**

The online version of this article (doi:10.1186/s12862-017-0998-8) contains supplementary material, which is available to authorized users.

## Background

Many species are currently expanding their ranges as a response to increasing global temperatures under climate change [1]. Range expansions are known to have profound effects on the genetic composition of populations, regarding both neutral and adaptive genetic diversity [2–5]. Mutations, even deleterious ones, can surf the wave of the range expansion and reach high frequencies in newly established populations [6]. In addition, traits that act to increase species’ dispersal capabilities and population growth rates are selected for under range expansions due to spatial sorting [7, 8] and kin competition [9]. This may lead to higher dispersal rates [10, 11], larger dispersal distances [5] and higher effective fertilities [12] at the expanding front of species’ ranges due to microevolution. An increasing dispersal rate under range expansion will increase the invasion speed [5], but only if the species is able to adapt sufficiently rapid to novel local conditions (and assuming the absence of strong Allee effects [13]). The depletion of genetic diversity at the expanding range border due to iterated founder effects [3, 4, 14] could however be expected to limit the invasion speed as low genetic diversity will lead to lower rates of local adaptation and thereby delayed population establishment [15, 16]. The surprising ability of colonizing species to adapt to novel conditions while being subjected to genetic bottlenecks is termed ‘the genetic paradox of invasive species’ [17].

Several possible explanations for ‘the genetic paradox of invasive species’ are reviewed in Stapley et al. [18], particularly highlighting the role of mutational processes as a source of new genetic diversity, and focusing on transposable elements. Another, more classic example of a mutational process is the evolution of mutation rates. Here selection can act on allelic variation in the processes of DNA repair and replication and as such result in increased mutation rates. These cause higher levels of genetic diversity and can thus enable adaptation to changing selection pressures [19–26]. However, the rate at which mutations occur is not a phenotype and thus cannot be selected for. Since selection acts on the mutation that occurs at a gene under selection and thus not on the rate at which such mutations occur, the establishment of a particular mutation rate is restricted to genetic hitchhiking. This is the phenomenon that an allele increases in frequency, because its locus is linked to another locus at which the allele is under positive selection. Usually this link is caused by physical proximity of the two loci, causing the different alleles to be inherited together. In this case, a genetic modifier that increases the mutation rate, increases in frequency by hitchhiking on beneficial mutations, and decreases in frequency by hitchhiking on deleterious mutations [27], inducing linkage disequilibrium (LD). Two loci are said to be in LD when their alleles are more (or less) frequently associated than can be expected under the assumption that they are independently inherited. When the genetic modifier produces a beneficial mutation, this modifier is likely to be passed on to the next generation because the beneficial mutation and the genetic modifier occur in the same individual, causing such LD. Recombination can however easily break up this joint inheritance and thus the linkage disequilibrium. Theoretical studies have thus concluded that in sexual populations, where there is recombination, the effect of beneficial mutations on the evolution of mutation rates is negligible [22] to small under changing environments [28, 29]. However, Johnson [27] has extended these studies by assuming a series of unique beneficial mutations. This increases the strength of linkage disequilibrium between the modifier locus and the mutation locus, due to constant recurring events of genetic hitchhiking. He showed theoretically that under these conditions, beneficial mutations can play a role in determining the evolutionarily stable mutation rate in sexual populations when environment conditions are constantly changing and costs of lower fidelity replication are low.

A species range consists of, more or less connected, separate populations of individuals, in contrast to the investigated single populations in previous theoretical studies [22, 27–29]. We argue that population structure, and in addition under range expansion, spatial sorting and iterated founder events, benefit the build-up and maintenance of linkage disequilibrium. Therefore, the evolution of mutation rate might be a considerable factor in the invasion ability of a sexually reproducing metapopulation. Here we investigate if the evolution of mutation rates could play a role in explaining the ‘genetic paradox of invasive species’ [17] for a sexually reproducing species colonizing a landscape with gradually changing conditions. We use a spatially explicit individual-based metapopulation model of a sexual species establishing its range on a spatial gradient to investigate 1) whether mutation rates increase during range expansion, 2) if this is related to the rate of dispersal during range expansion, and 3) if such increase of mutation rates is adaptive.

## Methods

We use a spatially explicit individual-based metapopulation model of a sexually reproducing species with discrete generations, which expands its range along a gradient in temperature. We allow the mutation rate to evolve, and investigate its interplay with the evolution of dispersal probability and temperature adaptation during and after range establishment.

### Landscape

The simulated landscape consists of 250 columns (*x*-dimension) of 20 patches each (*y*-dimension). We assume wrapped borders in *y*-direction, building a tube. Hence, if an individual leaves the world in *y*-direction during dispersal, it will reenter the simulated world on the opposite side. However, if it leaves the world in the *x*-direction, it is lost from the simulation. To answer our research questions the model requires a need for local adaptation during range expansion. Thus every column of patches (*x*-position) is characterized by its specific mean temperature τ_*x*_. This mean local temperature is used for the determination of the level of local adaptation of individuals. To simulate a large-scale habitat gradient, τ_*x*_ changes linearly from τ_*x*=1_ = 0 to τ_*x* = 250_ = 10 along the *x*-dimension, i.e. by Δτ = 0.04 when moving one step in *x*-direction.

### Population dynamics and survival of offspring

Local populations are composed of individuals, each of which is characterized by several traits: 1) its sex, 2) its dispersal probability determined by the alleles at the dispersal locus *l*_*d*_, 3) its optimal temperature τ_*opt*_, i.e. the temperature under which it survives best, determined by the alleles at its adaptation locus *l*_*a*_ (see below for details), 4) its genetic mutation rate determined by the alleles at the mutator locus *l*_*m*_ (see below under Genetics), and 5) a diploid neutral locus *l*_*n*_, for the sake of comparing the levels of genetic diversity with other loci.

Local population dynamics follow the time-discrete Beverton–Holt model [30]. Each individual female in patch *x*, *y* is therefore assigned a random male from the same habitat patch (males can potentially mate several times) and gives birth to a number of offspring drawn from a Poisson distribution with mean population growth rate λ. The offspring’s sex is chosen at random. Density-dependent survival probability *s*_1_ of offspring due to competition is calculated as:

$$ {s}_1=\frac{1}{1+\frac{\uplambda -1}{K}\cdot {N}_{x, y, t}} $$

with *K* the carrying capacity and *N*_*x,y,t*_ the number of individuals in patch *x,y* at time *t*. Finally, the surviving offspring experience a further density-independent mortality risk (1 − *s*_2_) that depends on their level of local adaptation, so the matching of their genetically determined optimal temperature (τ_*opt*_) to the temperature conditions in patch *x*, *y* (τ_*x*_) according to the following equation:

$$ {s}_2= \exp \left[\frac{-1}{2}\cdot {\left[\frac{\uptau_{opt}-{\uptau}_x}{\eta}\right]}^2\right] $$

where *η* describes the niche width or ‘tolerance’ of the species. We performed simulations for the species with a niche width of *η* = 0.5, equivalent to a decrease of survival probability of about 0.032 when dispersing one patch away from the optimal habitat. In this approach we assume that density-dependent mortality (1 − *s*_1_) acts before mortality due to maladaptation to local conditions (1 − *s*_2_). In addition, each population has an extinction probability ε per generation. Surviving offspring disperse with probability *d* that is determined by their dispersal locus (see below). If an individual disperses it dies with probability μ, which is 0.2 throughout the landscape. This mortality accounts for various costs that may be associated with dispersal in real populations, like fertility reduction or predation risk [31]. We assume nearest-neighbor dispersal, i.e. successful dispersers settle randomly in one of the eight surrounding habitat patches.

### Genetics

As mentioned above, each individual carries three unlinked, diploid loci coding for its dispersal probability, its optimum temperature (and thus its degree of local adaptation), and its genetic mutation rate, respectively, and an additional neutral locus. The phenotype of an individual is determined by calculating the means of the two corresponding alleles, with no dominance effect involved. Hence, dispersal probability *d* is given by *d* = (*l*_*d*,1_ + *l*_*d*,2_)/2 (with *l*_*d,*1_ and *l*_*d,*2_ giving the two ‘values’ of the two dispersal alleles), optimal temperature τ_*opt*_ is calculated as τ_*opt*_ = (*l*_*a*,1_ + *l*_*a*,2_)/2 (with *l*_*a,*1_ and *l*_*a,*2_ giving the ‘values’ of the two adaptation alleles), and similarly the mutation rate *m* = 10^-*exp.*^ (with *exp.* = (*l*_*m*,1_ + *l*_*m*,2_)/2, and *l*_*m*,1_ and *l*_*m*,2_ the ‘values’ of the two mutator alleles). At each of the four loci, newborn individuals inherit alleles, randomly chosen, from the corresponding loci of each of their parents. During transition from one generation to the next an allele at any locus may mutate with the genetically determined probability *m* given by the value based on the two alleles at the mutator locus *l*_*m*_ as elaborated above. So the mutator locus determines the mutation rate at each of the four loci. Mutations are simulated by adding a random number to the value of the inherited allele. This value is drawn from a Gaussian distribution with mean 0 and standard deviation 0.2. The lethal mutation probability Ω = 0.1 however, so 10 % of the mutations cause immediate death of the individual.

### Simulation experiments

Simulations were initialized with a ‘native area’ (from *x* = 1 to *x* = 50) from where the species was able to colonize the world, while the rest of the world was initially kept free of individuals. Upon initialization, dispersal alleles (*l*_*d,i*_) were randomly drawn from the interval 0 < *l*_*d,i*_ < 1, and mutator alleles *l*_*m,i*_ were set to 4, which set the initial mutation rate *m* to 10^−4^. Populations were initialized with *K* locally optimally adapted individuals, i.e. adaptation alleles were initialized according to the local temperature τ_*x*_. However, to account for some standing genetic variation we also added to every respective optimal temperature allele a Gaussian random number with mean zero and standard deviation 0.2. Identical copies of these alleles were used to initialize the neutral locus as well, for sake of comparison. We performed 200 replicate simulations, which all covered a time span of 15,000 generations. To establish equilibrium values, the individuals were confined to their native area during the first 10,000 generations. After this burn-in period, the species was allowed to pass the *x* = 50 border and expand its range for the remaining 5000 generations. Table 1 summarizes all relevant model parameters, their meanings and the standard values used for these simulations that test whether the evolution of mutation rates actually occurs.

**Table 1:** Parameter values

Parameter/variable	*(Initialization)* value	Meaning
*Individual variables*	evolving	
*l*_*d*,1_, *l*_*d*,2_	*0 to1*	alleles coding for the dispersal probability
*l*_*a*,1_, *l*_*a*,2_	*optimal with std. 0.5*	alleles coding for the optimal temperature
*l*_*m*,1_, *l*_*m*,2_	*4*	alleles coding for the mutation rate of the optimal temperature
*l*_*n*,1_, *l*_*n*,2_	*copy of l*_*a,1*_ *, l*_*a,2*_	neutral alleles as control
*Simulation parameters:*		
K	100	carrying capacity
λ	2	per capita growth rate
ε	0.05	local extinction probability
Ω	0.1	lethal mutation probability
μ	0.2	dispersal mortality
τ_x_	[0..10]	local temperature
η	0.5	niche width
x_max_	250	extent of simulated landscape in *x*-direction
y_max_	20	extent of simulated landscape in *y*-direction

As a follow-up, several other simulation experiments were performed to answer these specific questions:

1. Is the evolution of mutation rates neutral or adaptive? For this, the effect of evolving mutation rates on the speed of colonization was tested by contrast with fixed mutation rates. Therefore, the simulations were repeated with 200 replicates a) with fixed values of the mutation rate *m* of 10^−4^ and 10^−5^, combined with evolving dispersal probability, and b) with both values fixed, investigating the combination of *d* = 0.2 and *m* = 10^−4^, and of *d* = 0.2 and *m* = 10^−5^.
2. Does the mutation rate evolve in coevolution with the dispersal probability? For this, the dispersal probabilities were fixed to assess the effect on the evolution of mutation rates. The simulations were therefore repeated with 200 replicates for fixed values of dispersal probability, *d* = 0.05, *d* = 0.1 and *d* = 0.2, while allowing the mutation rate to evolve.
3. Can the mutation rate increase if the percentage of deleterious mutations is higher? For this a simulation experiment was performed with 90% lethal mutations, Ω = 0.9.
4. How does the evolution of mutation rates depend on directional selection? For this, a landscape was designed in which the temperature varies in the *x*-direction, to compare with the initial linearly increasing temperature gradient. We therefore performed an experiment with a variable spatial gradient in temperature. This required the application of a new habitat gradient, where τ_*x*_ still changes from τ_*x*=1_ = 0 to τ_*x* = 250_ = 10 along the *x*-dimension, but at each τ_*x*_ we added a random number in the range [−0.5, 0.5].
5. How does the evolution of mutation rates depend on the process of range expansion, i.e. a series of colonizations? For this, we looked into a stable, non-range expanding population to assess the dependency of such repeated colonization, for which we simulated a non-expanding species’ range under both temperature gradients, so applying a) a non-variable and b) variable temperature gradient in time (as defined in experiment 4). For these experiments the species was initialized in the whole landscape, so not restricted to the native area, and the temperature was steadily increased in the entire landscape. With this, equal selection pressure for the species was forced, but without a range expansion, by changing the temperature at the same rate as experienced by the marginal populations in the spatial scenarios. Global dispersal was applied here.

### Analysis

The individual phenotypes for the three traits were documented in time and space throughout the simulations and averaged per *x*-position. For the dispersal probability we calculated the arithmetic mean, while the mutation rate was averaged geometrically because the mutator gene codes for the exponent’s value. Genetic diversity was calculated as the variance in allelic values at the adaptation locus, the dispersal locus and the neutral locus, per *x*-position. The analysis of linkage disequilibrium was done using Genepop version 4.5.1 [32] testing for a significant association between diploid genotypes at two loci. We here test every pair of loci, consisting of the locus with the gene coding for mutation rate and all other loci, so pairs of *l*_*a*_ and *l*_*m*_ *, l*_*d*_ and *l*_*m*_ *,* and *l*_*n*_ and *l*_*m*_.

## Results

**Fig. 1:**
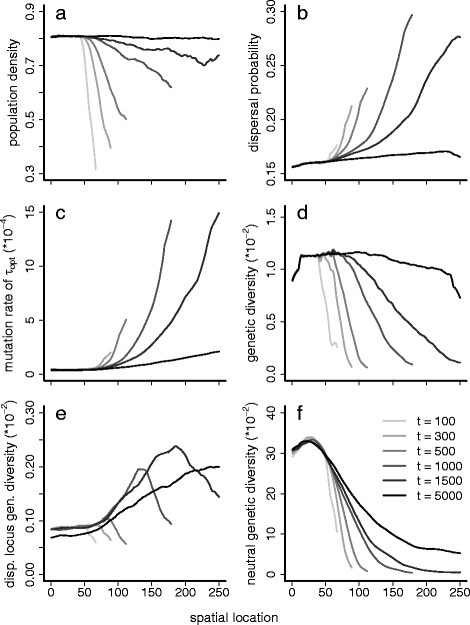
Base scenario. The average values over 200 simulations during and after range expansion across the gradient (*horizontal axis*) in time (*gray* *scaling from light to dark, as time proceeds, which is given in a sequence of generations 100, 300, 500, 1000, 1500, 5000*) of **a**. population density, **b**. dispersal probability, **c**. the mutation rate, **d**. genetic diversity at the adaptation locus, **e**. genetic diversity at the dispersal locus, and **f**. neutral genetic diversity, all measured as the variance in allele values. For reasons of clarity, a moving average with a window size of 21 has been applied (each point along the *x*-axis is the average of all points in the range [*x*-10, *x* + 10], data were present in 10-generation intervals)

The local level of adaptation *s*_2_ is close to one in all simulations, throughout the simulation time and across the entire species’ range. This indicates that colonization of new habitat occurs by pre-adapted individuals only. After the burn-in phase, the species’ range expands across the landscape (Fig. 1). The landscape is fully colonized after between 1000 and 1500 generations, after which the population density keeps increasing till it reaches 80% of the maximum overall (Fig. 1a). During the range expansion the average dispersal probabilities and mutation rates increase (Fig. 1b/c). This indicates that the individuals carrying alleles for high mutation rates are the first to establish new populations because they allow colonization by carrying these beneficial and novel alleles at the dispersal and temperature loci as well. After the colonization is complete and the range border has stabilized, mutation and dispersal probabilities decrease again (Fig. 1b/c). High dispersal probabilities are indeed only favorable with frequent population extinctions and low dispersal mortality [33]. Once the range border stabilizes, a low dispersal phenotype is more advantageous due to the assumed dispersal mortality. However, these slow dispersers by definition take some time to reach the area (genetic signature of range expansion, [34, 35]), especially when the mutation rate levels off and new dispersal phenotypes only slowly appear locally. The decrease of the mutation rate is caused by the processes at the adaptation (temperature) locus. Once the maximum level of genetic diversity has been reached here, and population densities are at their equilibrium values, more mutations cause maladaptation. The association with these deleterious mutations causes the modifier of increased mutation rate to decrease in frequency.

Genetic diversity at the different loci shows a typical pattern of range expansion (due to founder effects or spatial sorting) with little genetic diversity at the expanding range margin, which increases with the age of the populations (Fig. 1d-f). The maximum level of genetic diversity differs between the different loci (Fig. 1d-f), reflecting their different functions. At the neutral locus genetic diversity steadily increases, towards a maximum determined by population dynamics. At the gene that determines the individual’s optimal temperature the maximum genetic diversity is determined by the number of allele values that allow the individual’s survival at that particular local temperature.

**Fig. 2:**
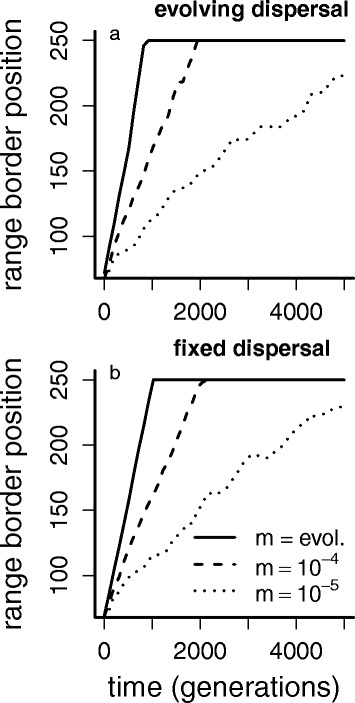
Experiment 1: fixed mutation rates. The range border position in time (*horizontal axis*) is shown, averaged over 200 simulations for the original experiment (with evolving dispersal probability) in panel **a** for the case with evolving mutation rate (‘control’) and fixed mutation rates of 10^−4^ and 10^−5^. Panel **b** is the same, but for a fixed dispersal rate of 0.2

**Fig. 3:**
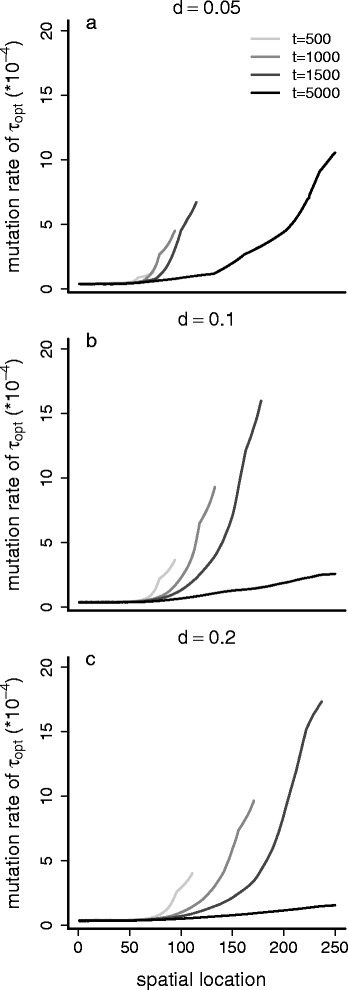
Experiment 2: fixed dispersal probabilities. The average values of the mutation rate during and after range expansion across the gradient (*horizontal axis*) is shown in time (*gray* *scaling from light to dark, as time proceeds, which is given in a sequence of generations 500, 1000, 1500, 5000*) under **a**. a fixed dispersal probability of 0.05, **b**. a fixed dispersal probability of 0.1, and **c**. a fixed dispersal probability of 0.2

To ensure that the observed increase in mutation rate is adaptive and not the result of reduced selection pressure at the range front, we performed the additional simulation experiments with fixed rates (experiments 1a and b). These show that a fixed, lower mutation rate (Fig. 2) causes a lower speed of invasion. This indicates that the observed evolving high mutation rate is indeed not neutral but beneficial, since it allows the faster occurrence of alleles coding for higher dispersal probabilities that establish on the expansion wave and increase the invasion speed. This is the same both for evolving dispersal probabilities (panel A) as for fixed dispersal probability (*d* = 0.2, panel B), so even if the dispersal probability is fixed to a relatively high value of 0.2, the expansion is slow if the mutation rate is no allowed to evolve. To assess how dispersal rates and mutation rates are connected we look into the results of experiment 2. Under fixed dispersal rates (Fig. 3) we see the evolution of higher mutation rates and faster range expansions with higher dispersal probabilities. This clearly indicates the coevolution of mutation rate and dispersal probability, with a similar advancing effect on the invasion speed due to the production of alleles at the gene for local adaptation to temperature, allowing the faster rate of local adaptation.

The analysis of linkage disequilibrium (Additional file 1: Figure S1) shows that LD is widespread across the species’ range, between every combination of loci, and also before and after the range expansion. Particularly at the onset of range expansion (*t* = 100, with *t* = 0 is the end of the burn-in period) there is a wide zone of newly colonizing populations at the expanding range margin with no genetic diversity at the temperature (i.e. local adaptation) locus (Additional file 1: Fig. S1a). A high initial population growth rate and the non-random set of invaders in such a newly established population result in a local non-random subset of the available genetic variation at the mutator locus. This causes strong linkage disequilibrium between all pairs of loci. The directional selection caused by the applied temperature gradient and the realistic metapopulation structure contribute to the maintenance and permanent renewal of LD.

The simulation experiment in which 90% of the mutations were lethal (Ω = 0.9, experiment 3) show a clear increase of the dispersal probability and mutation rate compared to the equilibrium conditions in the native area (Additional file 1: Figure S2B/C). Absolute values of both are however much lower than the values observed under Ω = 0.1, with the maximum of the mutation rate a factor 10 lower and the dispersal probability lower by a factor of almost 2. This has important consequences for 1) the invasion speed and population growth (Additional file 1: Figure S1A), with 5000 generations not being sufficient to colonize the whole landscape, and for 2) the pattern of genetic diversity at the dispersal locus (Additional file 1: Figure S1E), which is qualitatively different from the Ω = 0.1 simulations (Fig. 1e). The patterns in genetic diversity at the locus for temperature adaptation (Additional file 1: Figure S1D) and the neutral locus (Additional file 1: Figure S1F) only show quantitative differences with those in the base scenario of Ω = 0.1 in Fig. 1.

To determine the dependency of the evolution of high mutation rates on repeated colonizations we performed experiment 5. Here we omitted range expansion, applying a temporal temperature increase only, and added variation to the temperature gradient. For the spatially stable species’ range (experiment 5a and b, Additional file 1: Figure S3), a strong directional selection through a steady increase of the temperature induces an increase of dispersal probability (a, panel B) and mutation rate in time (a, panel C). Under variable temperature increase however (Additional file 1: Figure S3b) the mutation rate could not evolve (C) and the dispersal probability hardly changed (B), because the required linkage disequilibrium cannot build up. Overall the population densities in the stable species range are lower (panels A in Additional file 1: Figure S3a/b, ~50% compared to ~80% in the expanding range). Under variable temperature increase, population growth is additionally affected by the non-directionally changing selection pressure, resulting in variable population densities in time (Additional file 1: Fig. S3b, panel A). Under the same scenario of variably increasing temperature, but returning to the expanding population (experiment 4, Additional file 1: Figure S4), the mutation rate contrastingly did increase during colonization (Additional file 1: Figure S4C), as in all other range expanding scenarios. This indicates that a scenario of range expansion can replace a strong directional selection [27] in the build-up of linkage disequilibrium that is required for the evolution of high mutation rates. The other panels of Additional file 1: Figure S4 show qualitatively the same patterns as under the original scenario of steadily increasing temperature across the landscape (compare with Fig. 1). The important difference to notice however is in the absolute level of genetic diversity at the adaptation (temperature) locus (Additional file 1: Figure S4D), which is much higher under variable temperature increase, because more temperature values are now required to maintain locally adapted. Comparing Fig. 1d and Additional file 1: Figure S4D additionally shows that mutation rates above a certain threshold do not add more genetic diversity at the neutral and local adaptation loci. This is in contrast to the dispersal locus, where a higher mutation rate causes a higher level of genetic diversity.

## Discussion

In this study we investigate whether the evolution of mutation rates under the range expansion of a sexual species that needs to adapt to novel local temperature conditions can be an explanation for the ‘genetic paradox of invasive species’ [17], i.e. the ability of colonizing species to adapt to novel conditions while being subjected to genetic bottlenecks. We observe an increase of the mutation rate, which leads to a faster evolution of dispersal probability, faster adaptation to novel local temperature conditions, and thus faster range expansion. This also occurs when we apply variance to the mean temperature gradient in space, and even when we assume that 90% of the mutations are lethal. A simple analytical approach (Additional file 2) shows the increased fitness of individuals with higher mutation rates under large environmental changes, indicating that a stronger change in the environmental conditions should favor higher mutation rates in order to maximize the populations’ fitness expectations. The simulation experiments with fixed dispersal probabilities (so non-evolving, Fig. 3) clearly indicate that when dispersal probability is allowed to evolve (in Fig. 1), it coevolves with the mutation rate: the mutation rate only reaches really high levels when the dispersal rate is high as well. So a high mutation rate allows the dispersal rate to evolve to high values, but the high dispersal rate then requires faster local adaptation, indirectly selecting for even higher mutation rates. The genetic modifier that increases the mutation rate can increase in frequency by hitchhiking on beneficial mutations, despite the independent inheritance of the three traits in sexual populations. This result is particularly interesting as selection for optimum mutation rate is mostly associated with asexual populations [20–22, 26]. Indeed, selection only operates on the dispersal and temperature loci, favoring mutations that increase the speed of range expansion. In sexual populations, strong linkage is required for the (advantageous) alleles at the these loci and the (high) mutation rate allele at the mutator locus to be inherited together, and as such to lead to indirect selection at the mutator locus [26, 27, 36]. In our study, however, all loci are genetically unlinked, but linkage disequilibrium (LD) between all pairs of the different loci is widespread, with LD build-up aided by spatial sorting, and being maintained and renewed under population structure. Hitchhiking on deleterious mutations causes a decrease in the frequency of the genetic modifier that increases mutation rates at a later stage, when the colonization process is completed. In a stable species’ range, with the metapopulation not subjected to a range expansion but to a temporal increase of temperature, the mutation rate evolves as well, but not when variance is added to the mean temperature increase.

We have modeled the mutation rate as the probability that an inherited allele mutates. Since these mutation rates are caused by genes that are involved in processes of DNA repair and reproduction [23], high mutation rates will however likely affect the individual itself and not only its offspring as we modelled here. This effect is the result of mutations occurring when DNA is copied during the division of cells other than only the reproduction cells. Such mutations might then cause defects or tumors. Modeling mutation rates that negatively affect the individual’s fitness and not only its offspring will likely affect our results, because high mutation rates are then more disadvantageous [20, 21]. On the other hand, there are large differences in mutation rates between (parts of) genomes [37] and DNA repair is not restricted to a single pathway [38]. In addition, if a high mutation rate only affects an individual’s survival after reproduction, then high mutation rates might evolve despite their negative effects for the individual after it has completed reproduction.

Our results can be affected by the used genetic architecture, where linkage between traits [39, 40], polygeny, and the magnitude of mutations can be of importance in range dynamics [41–43]. The used mutation model of adding values to the inherited values result in mutations that are at most mildly deleterious at the adaptation locus, while an assumption of random mutations would invoke a stronger selection pressure [44]. However, our results are based on the assumption that 10 % of the mutations are lethal and we still see a significant increase of the mutation rate when we assume 90% lethal mutations (Additional file 1: Fig. S2). This is in contrast to what was found in an experimental study of sexual populations of yeast [45], which has however not taken a spatial perspective.

We observe that mutation rate can increase in combination with the increased dispersal probability and spatial variation, as experienced under range expansion. High dispersal rates, resulting in the immigration of many individuals, are expected to maintain a high local level of genetic variation [46], from which one would expect high levels of dispersal to be accompanied by a low local mutation rate. At the margin, however, relatedness amongst individuals increases at an advancing range front [9], reducing both local genetic diversity and the diversity of immigrants. Under these conditions an increase in the mutation rate evolves, which allows faster adaptation to the spatial variation in local temperature, causing a faster range expansion across the spatial gradient.

Holt et al. [46] investigated niche evolution at species’ range margins and found that local evolution is hampered when source populations of immigrating individuals are at low density, as a result of the stochastic processes in such populations [47–49]. Next to population density, the mutation rate also affects the probability of niche evolution in their study [46]: higher dispersal is limiting local evolution in the sink population under a higher mutation rate, because of the increased numbers of maladapted individuals from the source. Holt et al. [46] did, however, not allow the joint evolution of mutation rate and dispersal rate, but instead used fixed rates. As a result, the dispersal rate does not decrease after colonization, while the conditions in the sink population make its persistence dependent on the constant influx of (maladapted) individuals, both in contrast to the model presented here.

In our study we investigate the evolution of mutation rates. Dealing with novel environmental conditions or increased evolvability is however not restricted to mutation rates, but can be modeled in different ways, e.g. an increased magnitude of the phenotypic effect of mutations [50], an epigenetic effect, the evolution of modularity [51], degeneracy [52], or the evolution of generalism or plasticity [53]. Kubisch et al. [9] showed that when dispersal is a means of adaptation, by tracking suitable conditions during periods of change, genetic adaptation does not occur. Which kind of adaptation to change can be expected under specific ecological and environmental conditions is an interesting field of future investigation.

There is an ever-expanding pool of literature discussing the ecological and evolutionary dynamics of dispersal in the formation of species ranges [54]. While individual-based models have recently greatly extended our theoretical knowledge of interactions and evolution of traits during range expansion, empirical data have been restricted to a few well-known cases [5, 11, 12, 55]. Increasing ecological realism in our models [56] can improve the predictability of theoretical phenomena which can then be tested by data from field studies. So far, increased dispersal has been shown to increase invasion speeds [11, 57], affect the fate of neutral mutations [58], as well as the level of local adaptation [59], and local population dynamics [33], and in addition causes strong patterns of spatial disequilibrium [34, 35].

## Conclusions

In this study we show novel consequences of the particular genetic properties of populations under spatial disequilibrium, i.e. the coevolution of dispersal probability and mutation rate, even in a sexual species and under realistic spatial gradients, resulting in faster invasions. The evolution of mutation rates can therefore be added to the list of possible explanations for the ‘genetic paradox of invasive species’. We conclude that range expansions and the evolution of mutation rates are in a positive feedback loop, with possibly far-reaching ecological consequences concerning invasiveness and the adaptability of species to novel environmental conditions.

## Additional files

Additional file 1: Figures S1-S4. See supplementary file for full legends. **Figure S1.** Linkage disequilibrium. **Figure S2.** Experiment 3: 90% probability of lethal mutations. **Figure S3.** Experiment 5: no range expansion. **Figure S4.** Experiment 4: application of variation to the spatial temperature gradient. Analysis of linkage disequilibrium and extra simulations. (DOCX 671 kb)

Additional file 2: A simple analytical approach to calculate optimal mutation rates. Simple analytical approach showing fitness effects of different mutation rates (PDF 429 kb)
