# Supplementary material for: Evolving mutation rate advances the invasion speed of a sexual species
Source: BMC Evol Biol. 2017 Jun 26;17:150. doi: 10.1186/s12862-017-0998-8 (PMC5485585; doi:10.1186/s12862-017-0998-8)

Online Appendix A. Additional figures

**Figure S1. Linkage disequilibrium**
Analysis of linkage disequilibrium between the mutator locus and the three other loci. Per figure, each bar represents the full landscape of 20x250 habitat patches, at subsequent time steps of t = 100, t = 500, t = 1000, t = 1500, and t = 5000, being colonized by the species. Light green populations indicate significant linkage disequilibrium between each indicated pair of loci, and yellow populations have no genetic diversity (so a single allele only) at at least one of the two loci. The zone of such yellow populations marks the range border position.

a. LD between the *mutator locus* and the *temperature locus*



 *t = 100*

*

 t = 500*

*

 t = 1000*

*

 t = 1500*

*

 t = 5000*

*0* *250*
 *spatial location*

b. LD between the *mutator locus* and the *dispersal locus*



 *t = 100

 t = 500

 t = 1000

 t = 1500

 t = 5000*

c. LD between the *mutator locus* and the *neutral locus*



 *t = 100

 t = 500

 t = 1000

 t = 1500

 t = 5000*

**Figure S2. Experiment 3: 90% probability of lethal mutations**

The average values over 200 simulations during and after range expansion across the gradient (horizontal axis) in time (gray scaling from light to dark, as time proceeds, which is given in a sequence of generations 100, 300, 500, 1000, 1500, 5000) of A. population density, B. dispersal rate, C. mutation rate, D. genetic diversity at the adaptation locus, E. genetic diversity at the dispersal locus, and F. neutral genetic diversity, all measured as the variance in allele values for the scenario with 90% probability of lethal mutations. For reasons of clarity, a moving average with a window size of 21 has been applied (each point along the *x*-axis is the average of all points in the range [*x*-10, *x*+10], data were present in 10-generation intervals).


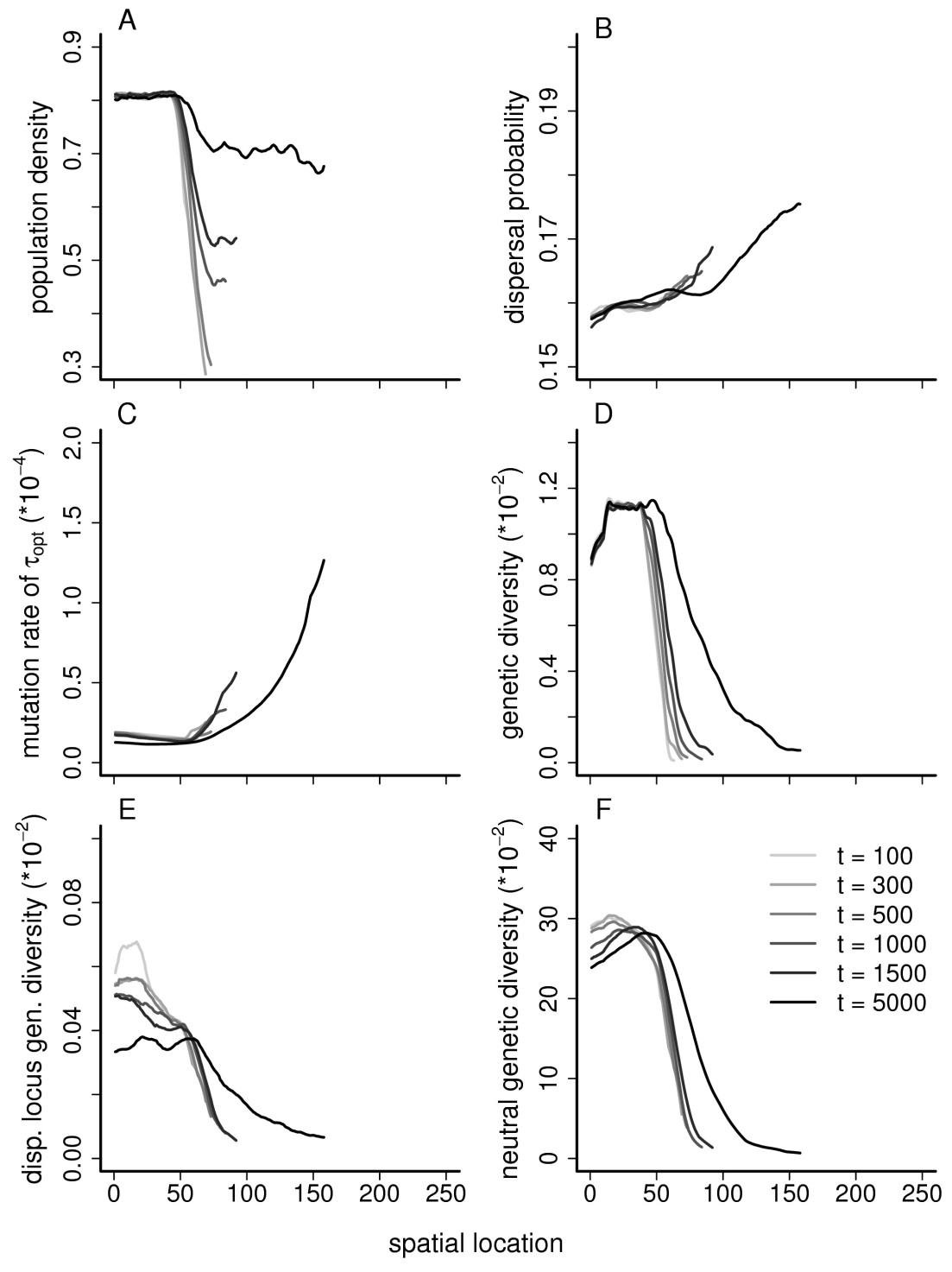


**Figure S3. Experiment 5: no range expansion**

The average values over 200 simulations in time in a spatially stable population subjected to a. a temporal linear gradient in temperature, and b. temporal variable gradient in temperature, of A. population density, B. dispersal rate, and C. the mutation rate. For reasons of clarity, a moving average with a window size of 21 has been applied (each point along the *x*-axis is the average of all points in the range [*x*-10, *x*+10], data were present in 10-generation intervals).

a.


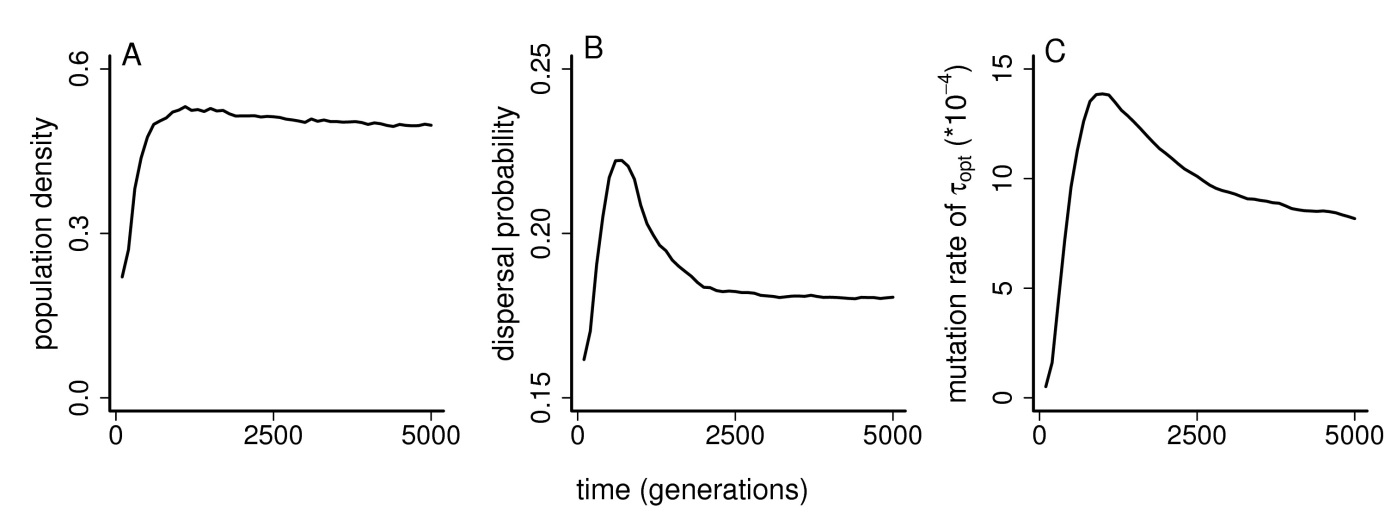


b.


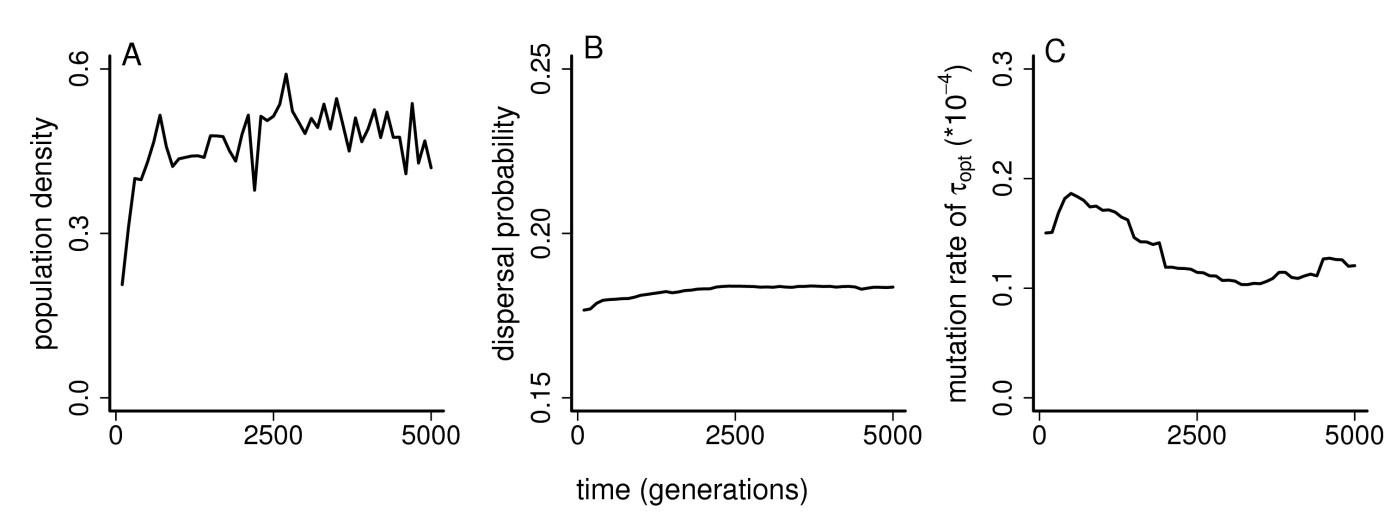


**Figure S4. Experiment 4: application of variation to the spatial temperature gradient**

Applying a variable spatial gradient in temperature, the average values are given over 200 simulations during and after range expansion across the gradient (horizontal axis) in time (gray scaling from light to dark, as time proceeds, which is given in a sequence of generations 100, 300, 500, 1000, 1500, 5000) of A. population density, B. dispersal rate, C. the mutation rate, D. genetic diversity at the adaptation locus, E. genetic diversity at the dispersal locus, and F. neutral genetic diversity, all measured as the variance in allele values. For reasons of clarity, a moving average with a window size of 20 has been applied (each point along the *x*-axis is the average of all points in the range [*x*-10, *x*+10], data were present in 10-generation intervals).


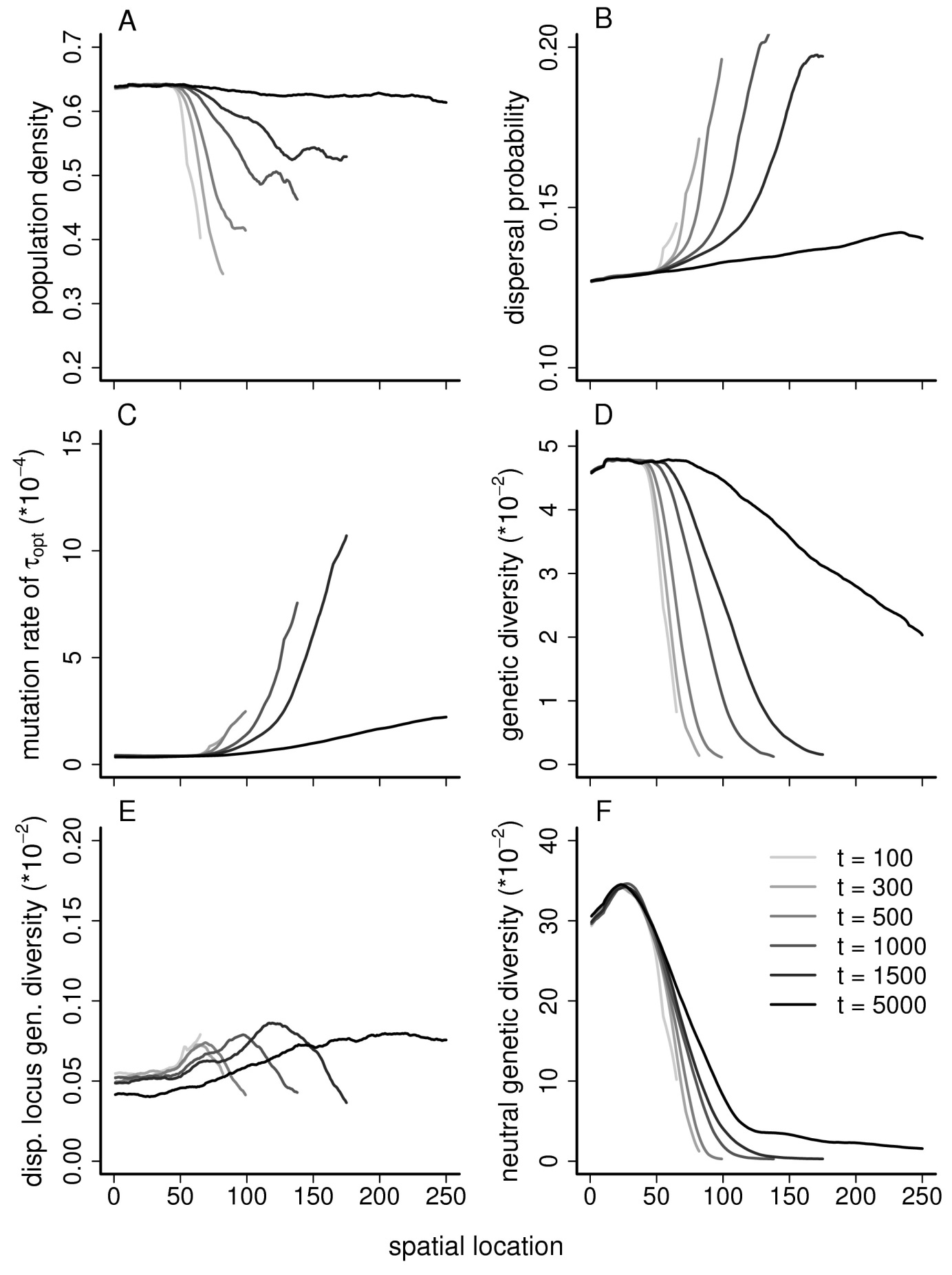

Supplement: Supplementary file 1 — See supplementary file for full legends. Figure S1. Linkage disequilibrium. Figure S2. Experiment 3: 90% probability of lethal mutations. Figure S3. Experiment 5: no range expansion. Figure S4. Experiment 4: application of variation to the spatial temperature gradient. Analysis of linkage disequilibrium and extra simulations. (DOCX 671 kb) [file 12862_2017_998_MOESM1_ESM.docx]
