# Supplementary material for: Evolving mutation rate advances the invasion speed of a sexual species
Source: BMC Evol Biol. 2017 Jun 26;17:150. doi: 10.1186/s12862-017-0998-8 (PMC5485585; doi:10.1186/s12862-017-0998-8)
Supplement: Supplementary file 2 — A simple analytical approach to calculate optimal mutation rates. Simple analytical approach showing fitness effects of different mutation rates (PDF 429 kb) [file 12862_2017_998_MOESM2_ESM.pdf]

A simple analytical approach to calculate optimal mutation rates

Cobben, M.P.P., Mitesser, O. & Kubisch, A.

The Model

We assume a very simplified model of selection for optimal environmental values, which we refer to as temperatures. We focus on a population living in this environment and calculate its fitness depending on occurring mutations of variable strength in the next generation.

Environment and fitness

Temperatures in the environment are denoted by τ_E. The effect of temperature on survival probability is assumed to be of Gaussian shape:

$$f(\tau_E) = exp\left(-\left(\frac{\tau_E - \mu_E}{s_E}\right)^2\right) \quad (1)$$

with μ_E denoting the environmental mean temperature and s_E the standard deviation of temperatures in the environment. Note that we assume that the curve scales to 1 to simplify further calculations, but could also be easily multiplied by an additional factur representing other temperature-independent survival components (Fig. 1).

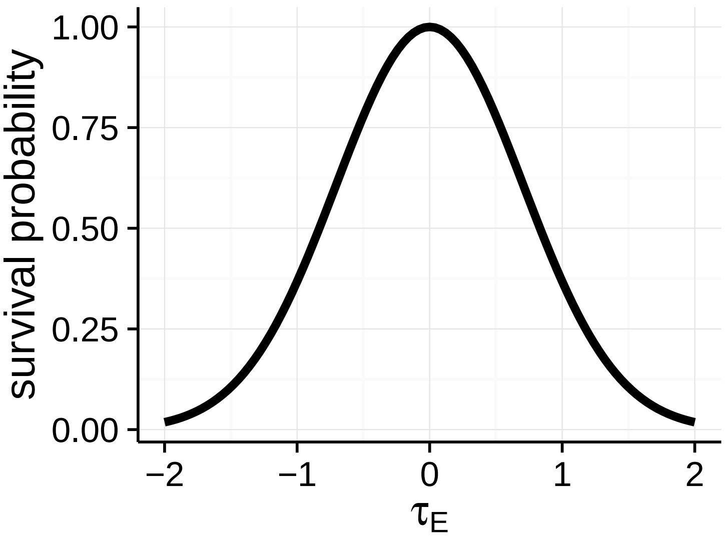

Figure 1: Survival probability in response to temperatures τ_E in the landscape. Parameter values are $\mu_E = 0$, $s_E = 1$.

Population

We further assume a population of individuals living in this environment, which is characterized by a distribution of trait values (i.e. individual optimal temperatures) that is also modeled as a Gaussian distribution with mean μ_P and standard deviation s_P:

$$g(\tau_P) = \frac{1}{s_P\sqrt{2\pi}} \cdot exp\left(-\left(\frac{\tau_P - \mu_P}{s_P}\right)^2\right) \tag{2}$$

Mutations

We assume that mutations, depending on their strength, affect the variability of the trait distribution at the population level. Assuming that the effect of mutations itself is Gaussian this results in an update of the population-level standard deviation s_P to s'_P:

$$s'_P = \sqrt{s_P^2 + s_m^2} \tag{3}$$

Environmental change

The environment changes to a certain degree in the next generation, the strength of which is denoted by Δ_τ. Here we assume no change in the variance, but only the mean of the distribution of temperature values in the environment (i.e. $\mu'_E = \mu_E + \Delta_\tau$).

Figure 2 shows the original (dotted gray line) and new effect of temperature on survival (solid black line) and the population's trait distribution following mutation (dashed red line) assuming a mutation strength of $s_m = 1.5$ and a change in the mean environment of $\Delta_\tau = 0.5$.

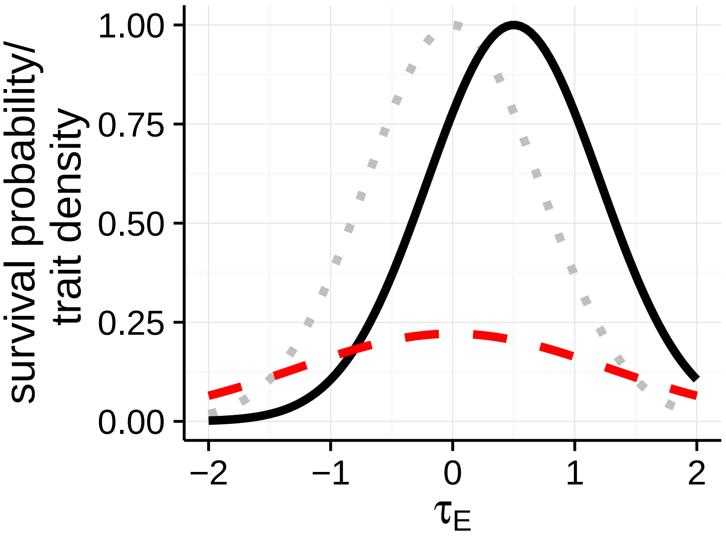

Figure 2: Survival probability in response to temperatures τ_E before (dotted grey line; environmental mean temperature: μ_E) and after environmental change (solid black line; environmental mean temperature: $\mu'_E = \mu_E + \Delta_\tau$). The dashed red line shows the trait distribution in the population after mutation. Parameter values are $\mu_E = 0$, $s_E = 1$, $\mu_P = 0$, $s_P = 0$, $s_m = 1.5$, $\Delta_\tau = 0.5$.

Mean fitness computation

To calculate the fitness effect of the given mutation strength we first calculate the product of above functions (i.e. $f(\tau) \cdot g(\tau)$), which gives us the survival probability for all occurring trait values:

$$h(\tau) = \frac{1}{s_P \cdot \sqrt{2\pi}} \cdot exp\left(-\left(\frac{\tau-\mu_P}{s_P}\right)^2 - \left(\frac{\tau-\mu_E}{s_E}\right)^2\right) \quad (4)$$

We integrate $h(\tau)$ over τ to calculate mean expected fitness ω:

$$\omega = \int_{-\infty}^{\infty} f(\tau)d\tau = \frac{1}{s_P \cdot \sqrt{2}} \cdot \frac{1}{\sqrt{s_P^{-2} + s_E^{-2}}} \cdot exp\left(-\frac{(\mu_P-\mu_E)^2}{s_P^2+s_E^2}\right) \quad (5)$$

The graph in Fig. 3 shows survival probability (black line) and fitness distribution (dashed red line) at the population level. The highlighted area below the trait distribution indicates expected mean fitness (calculated via integration, eq. 5).

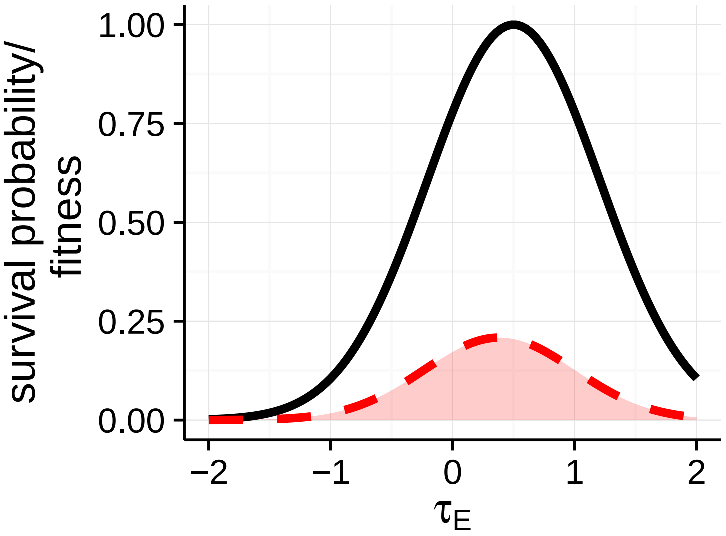

Figure 3: Survival probability in response to temperatures τ_E after environmental change (solid black line). The dashed red line shows the fitness distribution within the population. The highlighted area below the trait distribution indicates expected mean fitness (calculated via integration, eq. 5). Note that for reasons of clarity the original survival probability in response to temperatures before environmental change has been omitted. Parameter values as in Fig. 2.

Calculating optimal mutation rates

In order to calculate optimal mutation rates we simply create a vector of possible mutation rates for each strength of environmental change Δ_τ and compute their fitness effects using equation 5. We then pick the mutation rate (s_m), which maximizes the population's fitness expectations. Results are summarized in Figure 4: the left panel (A) shows (for four values of Δ_τ) the corresponding fitness values of each mutation strength. Red triangles indicate maximum values. The right panel (B) shows these optimal mutation rates as a function of the strength of environmental change Δ_τ.

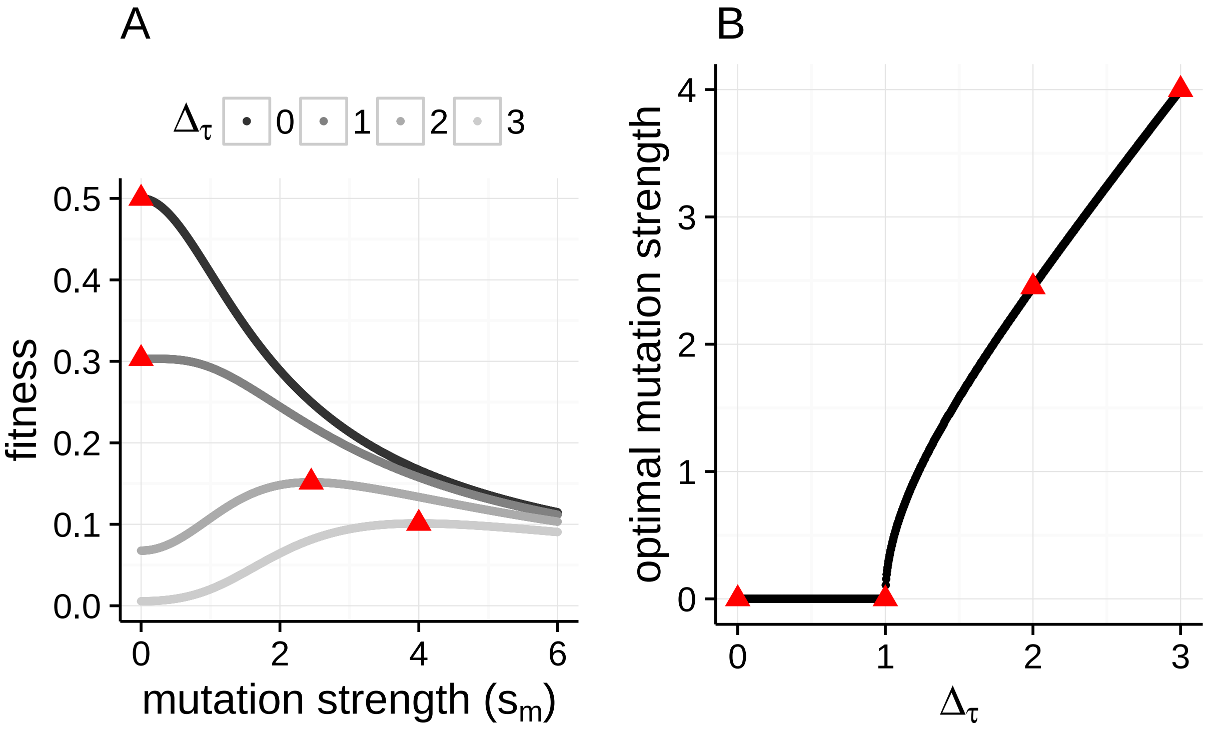

Figure 4: Results of the numeric approximation of optimal mutation rates. (A) The expected population fitness for given values of Δ_τ and mutation strength s_m. The red triangles denote the optimal values of s_m that maximize fitness. (B) The resulting optimal mutation strength plotted over the degree of environmental change Δ_τ. Parameter values: $s_P = 1$, $\mu_P = 0$, $s_E = 1$, $\mu_E = 0$.

Conclusions

Both the individual-based simulations from the main text and this simple analytical approach lead to the result that a stronger change in the environmental conditions should favor higher mutation rates in order to maximize the populations' fitness expectations. The analytical approach directly demonstrates that the combination of a normally distributed environmental characteristic and a corresponding normally shaped response in survival are sufficient to predict selection pressure on mutation rate.
